# Supplementary material for: A Winter-to-Summer Transition of Bacterial and Archaeal Communities in Arctic Sea Ice
Source: Microorganisms. 2022 Aug 10;10(8):1618. doi: 10.3390/microorganisms10081618 (PMC9414599; doi:10.3390/microorganisms10081618)
Supplement: Supplementary file 1 [file microorganisms-10-01618-s001.zip › Supplement S1.pdf]

Sample	Date	Latitude	Longitude	Floe	Season	Ice bloom
DNA01/RNA01	29-01-15	83,063	17,582	1	Winter	No bloom
DNA02/RNA02	05-03-15	83,142	24,1277	2	Winter	No bloom
DNA05/RNA05	12-03-15	82,9296	21,4409	2	Winter	No bloom
DNA07/RNA07	22-04-15	82,8478	16,5763	3	Winter	No bloom
DNA09/RNA09	21-05-15	81,2328	9,6369	3	Spring	No bloom
DNA10/RNA10	04-06-15	80,2939	4,02	3	Summer	Bloom
DNA11/RNA11	17-06-15	80,4759	7,8683	4	Summer	Bloom

Sample	Date	Bacterial and archaeal abundance [cells ml-1]	Ice thickness [cm]	Chl a concentratio n[mg m-3]	Nitrate[mol l-1]	Nitzschia frigida cell abundance [cells l-1]
DNA01/RNA01	29-01-15	3,54E+04	100	0,03	0,472	
DNA02/RNA02	05-03-15	2,40E+04	100		0,6	
DNA05/RNA05	12-03-15	1,77E+04	114	0,024	0,612	
DNA07/RNA07	22-04-15	8,78E+03	138,5	0,361	1,939	152062,2
DNA09/RNA09	21-05-15	6,35E+03	132	6,255		1297938,5
DNA10/RNA10	04-06-15	2,96E+04	122	3,148	1,427	716864,6
DNA11/RNA11	17-06-15	2,12E+04	92	1,036	0,918	

Sample	Date	Total Penales cell abundance [cells l-1]	Other eukaryotic cell abundance [cells l-1]	Total eukaryothic cell abundance [cells l-1]
DNA01/RNA01	29-01-15			
DNA02/RNA02	05-03-15			
DNA05/RNA05	12-03-15			
DNA07/RNA07	22-04-15	16308,9	21745,2	38054,2
DNA09/RNA09	21-05-15	548396,3	302908,0	851304,2
DNA10/RNA10	04-06-15	892863,6	520518,0	1413381,6
DNA11/RNA11	17-06-15			
